# Brain‐Derived Tau Oligomers in Circulating Extracellular Vesicles as Predictive Biomarkers for Alzheimer's Disease Conversion

**DOI:** 10.1002/alz70856_106869

**Published:** 2026-01-08

**Authors:** Michela Marcatti, Anna Fracassi, Wenru Zhang, Rakez Kayed, Giulio Taglialatela

**Affiliations:** ^1^ University of Texas Medical Branch, Galveston, TX, USA

## Abstract

**Background:**

Alzheimer's disease (AD) is one of the most common forms of dementia worldwide, making the identification of predictive biomarkers critical for early diagnosis and treatment during the preclinical stage. Recent studies have focused on blood‐based biomarkers, particularly plasma brain‐derived extracellular vesicles (pl‐BDEVs), to detect central nervous system (CNS) alterations. While blood biomarkers like amyloid proteins (Aβ42/Aβ40), tau, and phosphorylated tau show diagnostic promise, identifying predictive biomarkers remains essential. Blood total‐tau, originating from non‐brain sources, highlights the need to analyze brain‐derived tau (BDT) in pl‐BDEVs as a more specific biomarker for AD and other neurodegenerative diseases. Longitudinal studies offer great potential for detecting preclinical biomarker patterns in at‐risk individuals, yet little attention has been given to oligomers, the most toxic species in AD

**Method:**

In this study, we enriched pl‐BDEVs from CNS cell types from plasma samples longitudinally collected from participants enrolled in the Texas Alzheimer's Research and Care Consortium (TARCC), who were initially cognitively normal or displayed mild cognitive impairment (MCI), and later either progressed to AD (termed “converters”) or remained cognitively normal/MCI (termed “non‐converters”). We evaluated the isolated pl‐BDEVs by nanoparticle tracking analysis (size, number, and distribution), western blot (expression of extracellular vesicles markers: CD63, CD9, CD81), and electron microscopy. We immunoprecipitated brain‐derived tau oligomers (BDTOs) from pl‐BDEVs and characterized them by western blot, proteinase K (PK) digestion, seeding assay, and atomic force microscopy (AFM). Additionally, we treated SH‐SY5Y neuroblastoma cells and human synaptosomes isolated from cortex of control samples with these BDTOs and assessed their cytotoxicity and synaptotoxicity, respectively.

**Result:**

We demonstrated the successful isolation of pl‐BDEVs from plasma samples and the detection of BDTOs within these pl‐BDEVs. We identified distinct PK digestion patterns, morphology, and toxicity between BDTOs from converters and non‐converters. Additionally, our data revealed different distribution of BDTOs in the pl‐BDEVs isolated from the two groups. These findings suggest the presence of two different BDTO strains for converters and non‐converters.

**Conclusion:**

This study addresses the need for predictive AD biomarkers by analyzing previously unexplored BDTO conformers in pl‐BDEVs. Discovering distinct BDTOs in peripheral brain derived extracellular vesicles could enable preclinical forecasting and advance early‐stage AD treatments.

